# Pectoralis Major Tendon Tear in a 13-Year-Old Female High-Level Gymnastic Athlete: A Case Report

**DOI:** 10.7759/cureus.70343

**Published:** 2024-09-27

**Authors:** Johannes H van Ochten, Maxim Vanderstappen, Olivier Verborgt

**Affiliations:** 1 Orthopaedic Center Antwerp (Orthoca), AZ Monica, Antwerp, BEL; 2 Orthopaedics and Traumatology, University Hospital of Antwerp, Antwerp, BEL; 3 Medicine and Health Sciences, University of Antwerp, Antwerp, BEL; 4 Research Group Movement Antwerp (MOVANT) Department of Rehabilitation Sciences and Physiotherapy (REVAKI), University of Antwerp, Antwerp, BEL

**Keywords:** adolescent, diagnostic, pectoralis major, pectoralis major tendon tear, refixation, tear

## Abstract

Tears of the pectoralis major tendon have become more common due to the rising popularity of weightlifting and contact sports, yet they have not been described in young adolescents. A 13-year-old female high-level gymnastic athlete presented with left shoulder pain after a fall off the uneven bars. After excluding other diagnoses, magnetic resonance imaging (MRI) revealed a pectoralis major tendon tear. The tendon was surgically reattached using all-suture anchors via a deltopectoral approach. Postoperative care included using a shoulder immobilizer for four weeks, followed by a careful shoulder rehabilitation protocol. The recovery was uneventful.

## Introduction

Although tears of the pectoralis major tendon used to be rare, a large increase was seen over the last decades due to increased sports and fitness, especially in bench press and weightlifting activities. Not surprisingly, the highest incidence rates are observed in men between 20 and 40 years of age [1-3]. In gymnastics, however, a tear of the pectoralis major tendon has not been described in the literature. To date, only one case of a 23-year-old woman sustaining a pectoralis major tear has been described [4]. To our knowledge, a patient under 15 years old with a pectoralis major rupture has not been described in the literature until now [5]. 

The main function of the pectoralis major muscle, a powerful chest muscle, is adduction and internal rotation of the shoulder and some forward flexion of the arm. The pectoralis major muscle consists of a sternal head originating from the sternum and the first to sixth rib, and a clavicular head originating from the clavicle, forming a bilaminar tendinous insertion on the humerus just lateral to the bicipital groove. The clavicular head and superior three to five sternal segments form the anterior tendon layer, whereas the lower two to three sternal segments form the posterior layer [2, 6, 7].

The latest classification system of pectoralis major tendon tears was introduced in 2012 considering the bilaminar morphology of the tendon. Ruptures were divided based on the extent of the injury’s width and thickness, the timing of the injury, and its location. Ruptures were divided into complete or incomplete (width), full or partial (thickness), acute or chronic (timing), and according to the location of its origin (muscular part, the musculotendinous junction, or the tendinous insertion with or without bony avulsion) [1].

These injuries occur when the muscle undergoes maximal eccentric contraction with the arm in an abducted and extended position [8, 9]. Studies have shown that soft tissue avulsion of the pectoralis major at the humeral insertion is most observed [3]. Complete ruptures are more common than incomplete ruptures, whereas the sternal head is more frequently involved than the clavicular head of the pectoralis major tendon [3].

This report describes a traumatic pectoralis major tendon rupture including diagnostic, treatment, and rehabilitation modalities in a very young, female high-level gymnast without any predisposition. 

## Case presentation

A 13-year-old high-level female gymnastic athlete initially presented at the emergency department directly after trauma with a suspected shoulder dislocation after a dislodging trauma from the uneven bars and subsequent fall on the left shoulder. Upon presentation at the emergency department, a shoulder dislocation was clinically and radiographically excluded. A normal sling was given to prevent discomfort, and weight-adapted analgesia in the form of paracetamol and ibuprofen was administered. 

**Figure 1:**
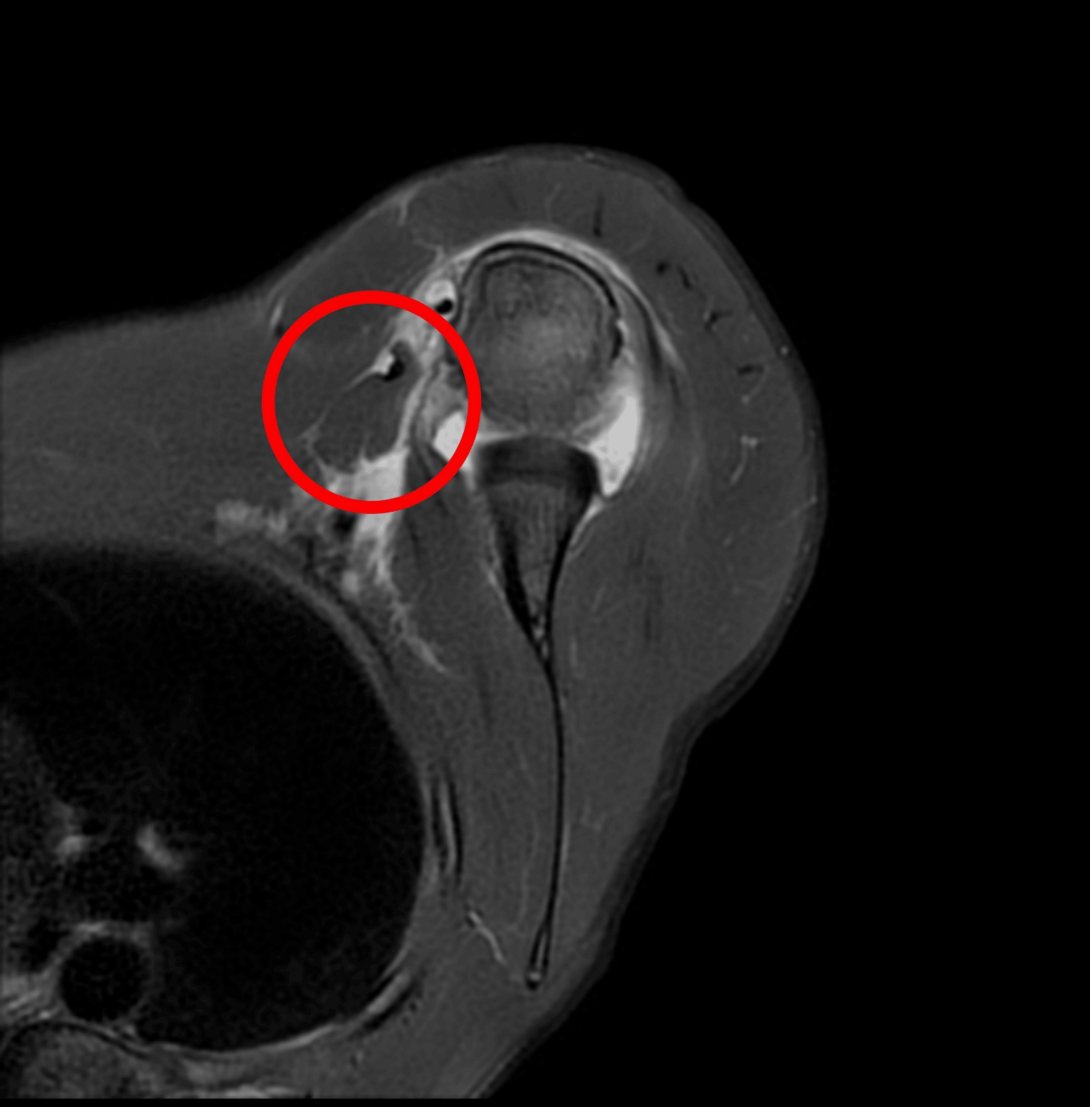
An axial MRI sequence shows a tear in the sternal head of the pectoralis major tendon.

The patient presented at our outpatient clinics 10 days after trauma with persistent complaints of pain in her left shoulder, which was still in the sling. Passively a full range of motion of the left shoulder was present, but an antalgic active limitation of range of motion was observed. Specific clinical shoulder tests like the Jobe test, lift-off test, belly-press test, and bear-hug test were negative, and none of the rotator cuff tendons showed any signs of weakness (Medical Research Council (MRC) 5 in all dimensions). Further diagnostic clinical testing revealed ecchymosis, persistent tenderness, and pain on the anterior side of the shoulder along the edge of the axillary fold, with a distinct weakness in adduction in combination with internal rotation (MRC 4). Magnetic resonance imaging (MRI) revealed a tear in the sternal head of the pectoralis major tendon (Figure 1). Especially because of the high demands of this patient, after careful consideration, explanation, and approval of her parents, operative treatment was scheduled. 

**Figure 2:**
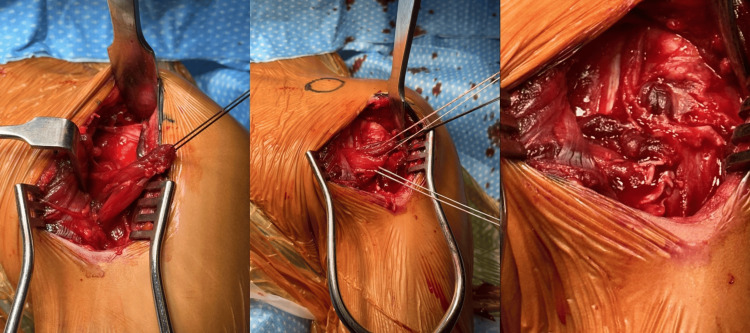
Surgical images a. The pectoralis major tendon's sternal head has been ruptured; b. The procedure involves placing a double-loaded all-suture anchor; c. The tendon is refixed using a double-loaded all-suture anchor.

Surgical exploration and refixation of the tendon were performed through a deltopectoral approach five days after visiting our outpatient clinics. During surgery, a small hematoma was encountered at the lateral side of the bicipital groove of the humerus, and a complete rupture of the sternal head was observed. Refixation was achieved using double-loaded all-suture anchors (Figures 2A-2C). Postoperative care included immobilization in adduction and internal rotation in a shoulder immobilizer for four weeks with passive mobilization up to 90 degrees of abduction and anteflexion, followed by active mobilization without weightbearing until the 10^th^ postoperative week [10]. A full range of motion was obtained after six weeks. 

## Discussion

As stated, an increase in tears of the pectoralis major muscle was seen in the last decades because of the growing popularity of fitness, especially weightlifting and bench-pressing. The use of anabolic steroids seems to be a risk factor [11]. Other risk factors include tobacco use, male gender, and use of specific antibiotics [2]. But as illustrated in our case, also in very young patients without any predisposing factors performing different kinds of sports, this injury should not be excluded in the differential diagnosis of traumatic shoulder complaints. 

Although rupture of the pectoralis major tendon is often a soft tissue tear, especially bony avulsions of the tendon have been described in adolescents in the literature [5,10]. However, we observed a soft tissue tear in our patient, which did not involve the bone. The extreme forces on the tendon experienced in high-level gymnastics could potentially explain this. We hypothesize that the moment of dislodgement from the uneven bars caused a very forceful eccentric contraction of the pectoralis major muscle, causing the tendon to tear. 

The diagnostic process begins with a thorough patient history. Patients usually feel a popping or tearing sensation, which was not experienced clearly by our patient, followed by pain and weakness. Physical examination in the acute setting can detect hematoma and ecchymosis, along with some palpable tenderness. One can detect subtle asymmetry of the axillary folds. Although the patient was not seen at the outpatient clinics directly after trauma, all of the described findings were still clinically detectable in our patient at the time of her visit. Additional radiographic diagnostics typically involve an MRI of the shoulder, which encompasses the pectoralis major tendon. However, most patients will receive a radiograph due to the complexity of the diagnosis and the high likelihood of overlooking injuries.

Treatment options are both conservative and operative, depending on patients’ demographics (age, demands, sports, etc.), although most of these injuries are treated operatively nowadays. Especially in high-level athletes, a well-functioning pectoralis major tendon is key for a good performance. Because of the high probability of missing these types of injuries, the risks are higher that additional grafts must be used to reconstruct the pectoralis major tendon. Usually, a direct refixation is possible in the acute phase until six weeks after injury. Beyond six weeks, the tendon tear becomes chronic and more difficult to reattach, although studies describe excellent outcomes of repair even after three months [2].

In the acute phase, several surgical techniques have been described to reattach the torn pectoralis major tendon. The most important techniques are transosseous, all-suture anchor, unicortical, and bicortical endobutton refixation, with most patients returning to sports after five to 10 months [11,12]. Two studies about surgical fixation techniques did not find any difference in failure rates between the techniques and advised the technique with which the surgeon is most familiar [13,14].

During postoperative treatment, patients are typically immobilized in a shoulder immobilizer for four to six weeks, depending on the strength of the fixation, following a careful shoulder rehabilitation protocol consisting of a protection phase until six weeks postoperatively, a motion phase until 10 weeks postoperatively, and a strengthening phase for the remainder as described by Shephard et al. [10].

## Conclusions

Tears of the pectoralis major tendon are rare but have become more common due to the increasing popularity of fitness activities (bench press and weightlifting). Although men between 20 and 40 years of age are most often involved, very young athletes performing different kinds of sports can experience this type of injury as well. Careful clinical and radiological examination leads to an early diagnosis, which in high-demand athletes is typically followed by surgical treatment. Specialized shoulder rehabilitation allows for full recovery of shoulder function and a complete return to sports activity.
